# Cell Differentiation of Bovine Milk Control Samples to Improve Prognosis of Mastitis Cure

**DOI:** 10.3390/antibiotics11020259

**Published:** 2022-02-17

**Authors:** Anne Bunge, Sonja Dreyer, Jan-Hendrik Paduch, Doris Klocke, Stefanie Leimbach, Nicole Wente, Julia Nitz, Volker Krömker

**Affiliations:** 1 Department of Microbiology, Faculty of Mechanical and Bioprocess Engineering, University of Applied Sciences and Arts, 30453 Hannover, Germany; anne@3wagners.de (A.B.); sonja.dreyer@outlook.de (S.D.); doris.klocke@hs-hannover.de (D.K.); stefanie.leimbach@hs-hannover.de (S.L.); nicole.wente@hs-hannover.de (N.W.); julia.nitz@hs-hannover.de (J.N.); 2 University of Cooperative Education, 08523 Plauen, Germany; jan-hendrik.paduch@ba-sachsen.de; 3 Department of Veterinary and Animal Sciences, University of Copenhagen, 1870 Frederiksberg C, Denmark

**Keywords:** differential cell count, mammary cure, somatic cell count, cell viability, bovine mastitis

## Abstract

To optimise udder health at the herd level, identifying incurable mastitis cases as well as providing an adequate therapy and culling strategy are necessary. Cows with clinical mastitis should be administered antibiotic medication if it is most likely to improve mammary cure. The somatic cell count (SCC) in milk of the monthly implemented Dairy Herd Improvement (DHI) test represents the most important tool to decide whether a cow has a promising mammary cure rate. Differential cell count (DCC) facilitates the specification of the immunological ability of defence, for example by characterising leukocyte subpopulations or cell viability. The aim of this study was to assess the DCC and cell viability in DHI milk samples regarding the cytological (CC) and bacteriological cure (BC) of the udder within a longitudinal study, thereby gaining a predictive evaluation of whether a clinical mastitis benefits from an antibiotic treatment or not. The cows enrolled in this study had an SCC above 200,000 cells/mL in the previous DHI test. Study 1 assessed the CC by reference to the SCC of two consecutive DHI tests and included 1010 milk samples: 28.4% of the mammary glands were classified as cytologically cured and 71.6% as uncured. The final mixed logistic regression model identified the total number of non-vital cells as a significant factor associated with CC. An increasing amount of non-vital cells was related to a lower individual ability for CC. Cows which were in the first or second lactation possessed a higher probability of CC than cows having a lactation number above two. If animals developed a clinical mastitis after flow cytometric investigation, the BC was examined in study 2 by analysing quarter foremilk samples microbiologically. Taking 48 milk samples, 81.3% of the mammary glands were classified as bacteriologically cured and 18.7% as uncured. The percentage of total non-vital cells tended to be lower for cows which were cured, but no significance could be observed. This study revealed that the investigation of the proportion of non-vital cells in DHI milk samples can enhance the prognosis of whether an antibiotic treatment of clinical mastitis might be promising or not. Prospectively, this tool may be integrated in the DHI tests to facilitate the decision between therapy or culling.

## 1. Introduction

Reducing the use of antibiotics in mastitis treatment requires a strict application only in bovine udders that have a promising cure rate. Therefore, the individual probability of being cured should be considered when assessing pathogen- and cow-related factors [1]. Although, an elevated somatic cell count (SCC) may be associated not only with udder diseases, but also with the stage of lactation or the season, e.g., the SCC is a useful indicator of intramammary infections (IMI) [2]. An udder is classified as incurable and chronically infected if the SCC exceeds 700,000 cells/mL in three consecutive Dairy Herd Improvement (DHI) tests or if the same pathogen is detected in three bacteriological tests in one lactation [3,4]. These cows should receive an antiphlogistic therapy and be culled at the most economically effective time [5]. To estimate the individual udder health status as precisely as possible, the count of leukocyte proportions can reveal the mammary ability to defend against invading pathogens [6]. Researchers such as Hageltorn and Saad [7] have already analysed leucocyte populations by flow cytometry and found differences in the differential cell count (DCC) of healthy and diseased udders. Since then, flow cytometry has emerged as an important diagnostic tool, providing specific investigations such as the estimation of cell viability or the specification of subpopulations by antibody labelling [8]. The leucocyte distribution in healthy bovine udders shows great variation in current studies. While some authors describe lymphocytes as being the dominant cell type, with 43–59% [9,10], other studies find macrophages to be the major cell population, with 35–79% [11,12]. If a pathogen invades the udder tissue, an immunological reaction starts and the polymorphonuclear leukocytes (PMN) migrate into the tissue and milk. Hence, the mean percentage of PMN increases during an early inflammatory response, often accounting for up to 90%, while the mean percentage of lymphocytes decreases [13,14]. The proportion of macrophages tends to decrease with increasing SCC [15,16]. In chronic bovine mastitis, the DCC in milk indicates an increase in the mean percentage of macrophages [7,15], while the main type of cells recruited into the mammary gland in the course of a persistent inflammatory accumulation are PMN [15,17]. The viability of bovine milk leukocytes, especially PMN and macrophages, provides valuable information about the immunological response of the mammary gland. Several studies suggest that an IMI is associated with an increase in PMN and higher cell viability than that of uninfected udders [13,18]. These previous findings recommend that low milk PMN viability could be considered as a risk factor for mastitis [19]. The objective of the present study was to evaluate the amount of highly granulated cells and the proportion of non-vital cells in DHI milk samples in order to predict the efficiency of antibiotic therapy for mammary cure if a cow suffers from clinical mastitis after flow cytometric investigation. The results aimed to provide reliable clusters of differentiation between cows with a favourable prognosis of mammary cure and those with a poor prognosis.

## 2. Results

### 2.1. Study 1—Cytological Cure

**Figure 1:**
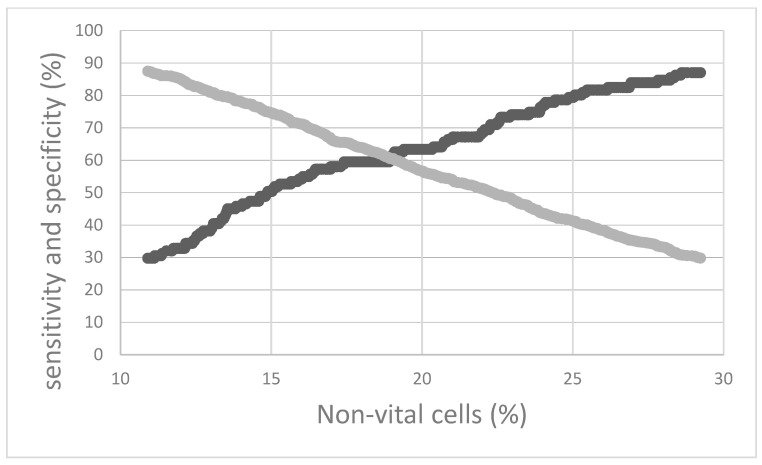
Sensitivity (dark grey) and specificity (light grey) of the proportion of non-vital cells for evaluating cytological cure.

In study 1, 1010 composite milk samples from 442 cows were included: 287 samples (28.4%) from 215 cows had an SCC below 200,000 cells/mL the month following the flow cytometric investigation, and 723 samples (71.6%) from 320 cows had an SCC above 200,000 cells/mL at the following DHI test. Hence, these mammary glands were defined as cytologically uncured. On average, the milk of mammary glands which were cytologically cured during the following month had 19.2% of total non-vital cells and 12.0% of highly granulated cells, and 30.3% of these highly granulated cells were not vital. The milk of cows without CC had a mean percentage of 24.6% of total non-vital cells and 13.1% of highly granulated cells, and 31.2% of these highly granulated cells were not vital. Cows having an amount above 18.9% of non-vital cells in milk were associated with a lower probability for CC than cows with an amount under this threshold, with a sensitivity and specificity of 60.3% and 60.6%, respectively (Figure 1).

**Figure 2:**
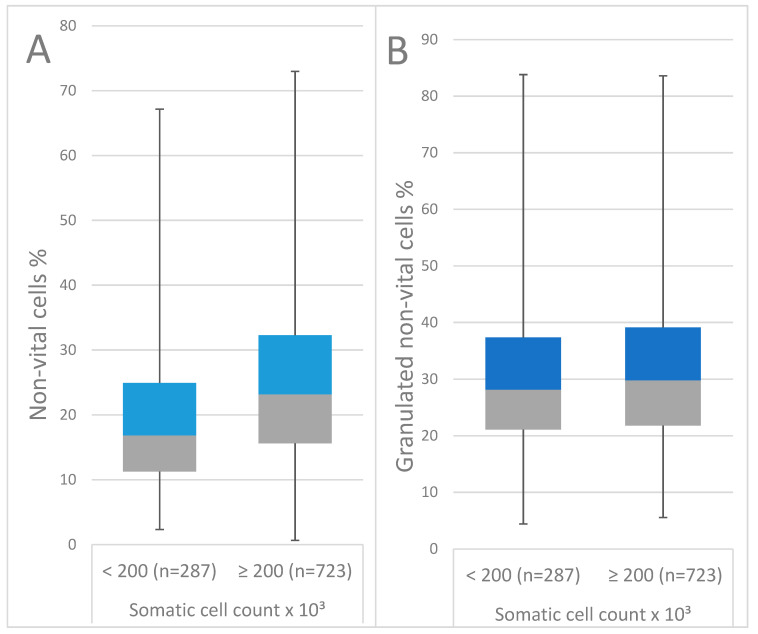
Box and whiskers plot of flow cytometric results of composite milk samples associated with the individual cytological cure: (**A**) percentage of total non-vital cells and (**B**) percentage of highly granulated non-vital cells.

The final mixed logistic regression model identified highly granulated non-vital cells (*p* = 0.001) and total non-vital cells (*p* < 0.001) as variables which are associated with CC. An increasing amount of total non-vital cells was related to a lower individual ability for CC of the udder (Figure 2).

The outcome for the proportion of highly granulated cells was not significant. The random cow within a herd effect which was included in the regression model displayed a significant effect on CC (*p* = 0.011). Cows which were in the first or second lactation had a significantly higher (*p* < 0.001) probability of CC than cows in the third lactation or higher (Table 1).

### 2.2. Study 2—Bacteriological Cure

The actual bacteriological cure (BC) was assessed for 48 DHI milk samples from 42 cows. The microbiological results of the mastitis and control samples were classified in accordance with the aforementioned definitions, yielding 9 samples (18.7%) from 9 bacteriologically uncured cows and 39 samples (81.3%) from 36 bacteriologically cured cows. The samples deriving from quarters which were bacteriologically cured included 48.7% new IMI after the mammary cure. Milk from bacteriologically uncured cows had an average percentage of 25.7% of total non-vital cells and 11.4% of highly granulated cells, with 32.3% of these highly granulated cells not being vital. If the cows were cured, the milk samples displayed an average percentage of 24.2% of total non-vital cells and 12.5% of highly granulated cells, with 36.1% of these highly granulated cells not being vital.

**Figure 3:**
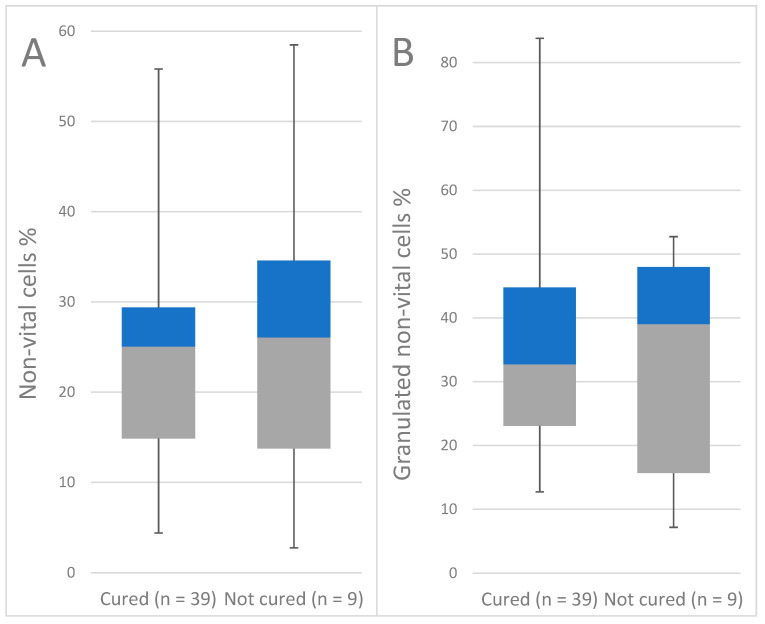
Box and whiskers plot of flow cytometric results of composite milk samples associated with the individual bacteriological cure: (**A**) percentage of non-vital cells and (**B**) percentage of highly granulated non-vital cells.

The final mixed logistic regression model could not observe a significant influence on the BC of any variable which was taken into account. The results are shown in the descriptive statistics (Figure 3). 

The microbiological examinations of the mastitis milk samples identified *Streptococcus (Sc.) uberis* as the most cultured pathogen (20.8%), followed by coliform bacteria, *Escherichia (E.) coli* and non-*aureus*-staphylococci (NAS) (Table 2).

## 3. Discussion

This flow cytometric investigation assessing differential cell count and cell viability in bovine DHI samples focused on the CC and BC of mammary glands which had an SCC above 200,000 cells/mL the month before the flow cytometric examination. Cows with clinical signs of acute mammary inflammation were excluded from the milking within the DHI test. The flow cytometric results aim to provide a prognosis of whether a diseased mammary gland may benefit from antibiotic therapy or not. 

Indeed, there are several technical causes of cell preparation and flow cytometric analysis which interfere with the test accuracy. As this tool was included in the DHI investigations, where analyses are performed on thousands of samples every day, the cell preparation needed to be rapid. That is why no adjustment of cell concentration for a higher precision of measurement took place. Milk sampling and storage in plastic tubes decrease the flow cytometric assessed amount of PMN and especially macrophages, because phagocytes adhere to abrasive surfaces [20]. Likewise, using composite milk samples instead of quarter milk samples has a great impact on flow cytometric results. Thus, the cell properties in milk of the diseased quarter are diluted with milk from the neighbouring quarters. However, the cell influx and viability in milk of uninfected quarters is influenced by the neighbouring diseased quarter, although these studies were not conducted on chronic mastitis cases [12,19]. According to the research of Merle et al. [12], who found cell viabilities in milk of uninfected quarters to be almost as high as in milk of the neighbouring diseased quarter, it may be suggested that the dilutive effect has a lower impact on the assessed percentage of non-vital cells than on the proportion of highly granulated cells [21]. The interdependence of mammary gland quarters was also confirmed via the assessment of the transcriptional response. Uninfected mammary gland quarters of neighbouring diseased quarters express genes associated with mammary infection. Thereby, the strength of the transcriptional response is dependent on the pathogen type [22]. According to [22], it may be presumed that the DCC and cell viability in neighbouring uninfected quarters depend on the infectious pathogen. This study analysed the cows’ individual probability for mammary cure. However, the significant random cow within a herd effect of the statistical model indicates that individual cow and environmental effects have a great impact on the DCC and cell viability. For example, a negative energy and nutrition balance impairs the migration of leucocytes into the mammary tissue by high ketone plasma concentrations [23].

Our results revealed an association between the proportion of non-vital cells in milk and the CC of the animal. Higher amounts of non-vital cells are significantly associated with a lower probability of CC. A negative impact of apoptotic cells for mammary cure is confirmed by numerous studies (e.g., [9,24,25]). Apoptotic PMN have a decreased bactericidal capacity, and furthermore they compete with pathogens to be phagocytised by macrophages [9,24]. Regarding this study, the resolution of inflammation as well as the reasons for its failure are of special interest. Apparently, 72% of our examined milk samples derived from cows did not cure cytologically. This indicates that high amounts of non-vital cells are related to an impaired resolution of inflammation [26,27]. Sladek and Rysanek [18] confirm that the resolution of bovine mastitis is initiated by the apoptosis of PMN and their phagocytosis by macrophages. This process seems to be delayed by cytokine and bacterial products such as interleukin-6, interferon-γ, granulocyte-macrophage colony-stimulating factor (GM-CSF) or lipopolysaccharide (LPS) [27,28]. Delayed apoptosis of PMN represents an immunological pattern, increasing the number of phagocytising PMN for an effective pathogen elimination. Migrated immunocompetent macrophages phagocytise early apoptotic PMN before they can release their granule contents (containing enzymes such as lysozymes, collagenase, gelatinase, histaminase, elastase and plasminogen activators), which would result in massive tissue damage and promote persistent inflammation [29,30]. If the amount of apoptotic PMN exceeds the phagocytic capacity of macrophages, PMN are considered to die through secondary necrosis, releasing toxic contents and inhibiting resolution [31,32]. The lipid mediator lipoxin A_4_ can enhance the phagocytic capacity of macrophages and suppress PMN chemotaxis [33,34]. The authors of [15] verified low lipoxin A_4_ concentrations in chronic infected mammary glands and suggested a relationship with non-resolving mastitis. For measuring non-vital cells, the dye propidium iodide was used, which stains the DNA of late apoptotic and necrotic cells [35]. Contrary to early apoptotic cells, these assessed cell stages have a high risk of releasing granule contents into the extracellular compartment [31]. Therefore, the results support previous findings that tissue damage caused by granule content release is associated with persistent non-resolving inflammation [30].

The detection of clinical mastitis and its microbiological examination were conducted in order to provide a prognosis for BC from the DCC in milk. Due to the former elevated SCC, the sampled cows were considered to have already experienced a mammary infection which became subclinical or chronic. Thus, the DCC was detected during a status without symptoms of acute mammary inflammation. The evaluation of DCC in relation to the BC did not provide significant results. A larger sample size with a differentiation of the mastitis-causing pathogen may have optimised the outcome. Several studies describe an influence of the number and type of bacteria on the DCC and cell viability in milk [36,37]. Recently, the host–pathogen interaction has gained better understanding since mammary epithelial cells have been identified as crucial for the first pathogen recognition and initiation of the immune response. Thereby, prominent differences in the response of mammary epithelial cells to *E. coli* or *Staphylococcus (S.) aureus* were detected. Whereas *E.coli* induces a very strong expression of cytokines and bactericidal factors in mammary epithelial cells, *S. aureus* displays a delayed response [38,39]. The pathogen elimination and resolution of inflammation is crucially determined by the pathogen-dependent activation of immune functions of the mammary epithelial cells [40]. Further examinations should be obtained to identify pathogen-dependent leukocyte or viability patterns and examine whether they are capable of prognosing BC.

## 4. Materials and Methods

### 4.1. Definitions

Cytological cure (CC): A quarter was considered as cytologically cured if the SCC of the month following flow cytometric analysis was less than 200,000 cells/mL.

Bacteriological cure: A quarter was defined as bacteriologically cured if the pathogen of the mastitis milk sample was not detected in the control sample or if a new isolate was detected in the control milk sample indicating a new IMI. Animals without clinical cases of mastitis in the course of the trial were not treated with antibiotics. Animals with clinical mastitis received local treatment with beta-lactam antibiotics; here, with amoxicillin and clavulanic acid.

No bacteriological cure: A quarter was considered uncured if the same pathogen was isolated in the control milk sample as in the mastitis milk sample.

### 4.2. Animals and Milk Sampling

This study was conducted from August 2014 until July 2016 on two commercial dairy farms in Northern Germany which participated in the monthly DHI sampling. The dairy farms milked approximately 550 and 1500 Holstein-Friesian cows twice a day in milking parlours. The mean individual animal yields were 10,800 and 11,500 kg ECM. The cell contents of the bulk milk were constantly below 150,000 cells/mL in both farms during the trial period. The animals were kept in loose housing. A lime/straw mixture was used as bedding material, which was supplemented every two days. In addition to manual pre-milking, the milking process included pre-disinfection, cleaning with wipes (1 per animal), post-disinfection and intermediate disinfection of the milking equipment. 

For this study, we analysed composite milk samples of dairy cows which had an SCC above 200,000 cells/mL in the latest DHI test. This threshold was chosen to increase the specificity of the identification of diseased mammary glands [41]. Cows with clinical signs of an acute intramammary inflammation were excluded from sampling, as well as cows which had not been sampled in the monthly DHI test on two consecutive occasions to assess their cytological cure. The selected animals were in different lactations and stages of lactations. The milk was sampled monthly during the normal milking process, where a compound of all milk fractions and all quarters per cow was collected in a sampling container. Approximately 30 mL of milk was transferred to 40 mL polyethylene plastic tubes, transported to the microbiology laboratory at the University of Applied Sciences and Arts (Hannover, Germany), stored at 6 °C and analysed within 18 h by flow cytometry.

Animals were included in study 1 if they showed a somatic cell count above 200,000 in the milk control and if they also participated in the DHI test in the two following months to assess their CC based on SCC. If cows developed clinical mastitis after flow cytometric analysis, they were also included in study 2. To determine BC, quarter foremilk samples were collected for bacteriological culture, one sample at the time of mastitis detection and a control sample 14–21 days after the end of treatment according to the standards of the German Veterinary Society [40].

The samples with the preserving agent Ly20 containing boric acid were stored below 8 °C and transported to the microbiology laboratory at the University of Applied Sciences and Arts (Hannover, Germany) within the following 5 days.

### 4.3. Somatic Cell Counts and Bacteriological Examinations

The SCC of the DHI milk samples for study 1 was observed in the milk laboratory of the State Control Association Weser-Ems (Leer, Germany) by the Fossomatic FC 500 (Foss, Hillerød, Denmark). Microbiological examinations were performed at the microbiology laboratory at the University of Applied Sciences and Arts (Hannover, Germany) in accordance with the guidelines of the German Veterinary Association, which are comparable to the National Mastitis Council recommendations [42,43]. Briefly, 10 µL of a well-mixed sample was streaked onto a quadrant of an esculin blood agar plate (Oxoid, Wesel, Germany). The plates were aerobically incubated at 37 °C and examined after 24 and 48 h. Colonies were identified by Gram staining, cell morphology, haemolysis patterns, esculin hydrolysis and the catalase test.

### 4.4. Differential Cell Count—Sample Preparation

The upper cream layer and lower sediments were mixed by gently shaking the samples. Afterwards, 10 mL of milk was transferred from the 40 mL polyethylene tube to the 13 mL sample tubes (Sarstedt AG and Co., Nümbrecht, Germany). The isolation of somatic cells was performed by centrifugation at 200× *g*, for 15 min at 4 °C. Afterwards, the cream layer and the supernatant were discarded and remaining fat residues on the inner tube wall were removed with a swab. The cell pellet was washed by resuspending it in 10 mL of phosphate buffered saline (PBS, Amresco, Solon, Ohio, USA) and centrifuged a second time at 200× *g* for 15 min at 4 °C. The supernatant was discarded again and subsequently, the cell pellet was resuspended in 2 mL of PBS and cooled at 4 °C until analysis. The DNA and RNA of the somatic cells were stained with acridine orange (AO) (Roth, Karlsruhe, Germany, final concentration 0.4 ng/mL) and propidium iodide (PI) (Roth, Karlsruhe, Germany, final concentration 3 µg/mL). Then, 2 mL of cell suspension and 30 µL each of AO and PI were filled into 3.5 mL test tubes (Sarstedt AG and Co.), which were incubated for 10 min and were resuspended before analysis.

### 4.5. Differential Cell Count—Flow Cytometric Analysis

The flow cytometer (CyFlow Space, Sysmex, Norderstedt, Germany) was equipped with a blue solid-state laser and a red diode laser with excitation wavelengths of 488 and 638 nm, respectively. The instrument was aligned and standardised with calibration beads (Beats Mix, Sysmex, Norderstedt, Germany) at the beginning of an examination. The software Flow Max was used for signal recording and analysis. After a live gate on green fluorescent particles had been set in order to disregard non-cellular events, 20,000 events were acquired. The AO-positive particles (536 nm band pass filter) were plotted on a forward light scatter (FSC) versus a side light scatter (SSC) dot plot. A gate on highly granulated cells was added to the dot plot. Measuring the proportion of non-vital cells, the green fluorescent cells were assessed for their PI positivity (675 nm band pass filter), whereas highly fluorescent particles were evaluated as non-vital cells. The proportion of non-vital cells of all AO-positive particles and of highly granulated cells was analysed in two dot plots.

### 4.6. Statistical Analysis

The data were collected with Excel 2013 (Microsoft Corporation, Albuquerque, NM, USA) and analysed with SPSS (IBM SPSS 26.0.0.0, Armonk, NY, USA). A monthly observation of a cow represented the statistical unit. CC and BC represented binary dichotomous-dependent variables. CC and BC were defined according to the aforementioned definitions and encoded as 0 and 1, respectively. Outcomes were analysed using generalised linear mixed models, including the proportions of highly granulated cells, highly granulated non-vital cells, total non-vital cells and lactation number as factors and covariates. The lactation number of a cow was grouped as 1, 2 and >2, respectively. A random cow within a herd effect was included in the model. Statistical significance was assumed at α = 0.05. For the final regression model, the linear predictor was given by:

Logit (outcome) = % highly granulated cells + % highly granulated non-vital cells + % non-vital cells + lactation number + herd (random) + e.

Thresholds as well as their sensitivity and specificity were assessed by receiver operating characteristic (ROC) curve analysis.

## 5. Conclusions

This study revealed that the amount of non-vital cells in DHI milk samples was able to improve the prediction of the individual probability of mammary cure. It represents an additional reference to decide whether clinical mastitis should be administered further antibiotic medication or not. Prospectively, this tool may be integrated in the DHI investigation and support the prognosis of mammary cure, together with common tools such as the SCC evaluation. Further investigations should be conducted in order to prove DCC and cell viability as being a predictive tool for BC. The current study outlines that the attempt to reduce the time of cell preparation for flow cytometric investigations may result in a loss of test accuracy. Therefore, the development of a fast and accurate flow cytometric procedure for use in DHI investigations will be challenging.

## Figures and Tables

**Table 1:** Final mixed logistic regression model for the individual ability of cytological cure.

	Coefficient				
Variable	X	SE ^1^	OR ^2^	95 % CI ^3^	*p*-value
Intercept	0.597	0.236	1.816	1.144–2.883	0.011
Highly granulated cells %	−0.002	0.011	0.998	0.976–1.020	0.839
Highly granulated non-vital cells %	−0.024	0.007	0.976	0.962–0.991	0.001
Total non-vital cells %	0.062	0.009	1.064	1.045–1.083	<0.001
Lactation number 1	−1.002	0.222	0.367	0.237–0.568	<0.001
Lactation number 2	−0.687	0.188	0.503	0.348–0.727	<0.001
Lactation number > 2 (reference)	0				

^1^ Standard error. ^2^ Odds ratio. ^3^ Confidence interval.

**Table 2:** Bacteriological culture results of the mastitis milk samples (n = 48).

Microorganism	Number	%
*Streptococcus uberis*	10	20.8
Coliform bacteria	9	18.8
*Escherichia coli*	7	14.6
NAS ^1^	7	14.6
*Enterococcus* spp.	3	6.3
Others	5	10.4
Mixed infections	7	14.5
Total	48	100

^1^ Non-*aureus*-staphylococci.

## Data Availability

The data presented in this study are available on request from the corresponding author. The data are not publicly available due to privacy.
